# Adaptive tight frame based multiplicative noise removal

**DOI:** 10.1186/s40064-015-1655-6

**Published:** 2016-02-12

**Authors:** Weifeng Zhou, Shuguo Yang, Caiming Zhang, Shujun Fu

**Affiliations:** School of Mathematics and Physics, Qingdao University of Science and Technology, Qingdao, 266071 Shandong China; School of Computer Science and Technology, Shandong University, Jinan, 250101 Shandong China; School of Mathematics, Shandong University, Jinan, 250100 China

## Abstract

Sparse approximation has shown to be a significant tool in improving image restoration quality, assuming that the targeted images can be approximately sparse under some transform operators. However, it is impossible for a fixed system to be always optimal for all the images. In this paper, we present an adaptive wavelet tight frame technology for sparse representation of an image with multiplicative noise. The adaptive wavelet tight frame is first learned from the logarithmic transformed given images, and then it is used to recover these images. Compared with the existing non-adaptive wavelet sparse transform methods, the numerical results demonstrate that the proposed adaptive tight frame scheme improves image restoration quality.

## Background

Noise is usually inevitable during data acquisition, transmission and record with CCD sensors etc. Image denoising as the most fundamental problem in image processing field is used to enhance images by reducing any degradations. Generally speaking, noise can be classified into additive noise and multiplicative noise based on the relationship of the noise and the signal. Unlike additive noise, multiplicative noise (e.g. speckle random noise) intensity is in proportion to the absolute image intensity and it mainly appears in synthetic aperture radar (SAR), laser imaging, ultrasound imaging, and positron emission tomography (PET) etc. (Goodman 2007; Oliver and Quegan 1998; Wagner et al. 1983) and has been paid great attention in recent years (e.g. Shi and Osher 2008; Aubert and Aujol 2008; Durand et al. 2010; Huang et al. 2012; Yu and Acton 2002; Steidl and Teuber 2010; Bioucas-Dias and Figueiredo 2010; Teuber and Lang 2012; Krissian et al. 2007). Mathematically, the degraded image *f* corrupted by multiplicative noise $\delta$ usually can be formulated as

1 $$\begin{aligned} f=u\cdot \delta . \end{aligned}$$

Techniques and algorithms have been developed for solving this inverse problem to obtain an approximate image $u^{*}$ to the original image *u* in recent years. Thereinto sparsity regularization methods are effective methods of them and have been widely used in every branch of image processing based on the hypothesis that images are approximately sparse under some transform domain *W*. Various linear operators that used to sparsely represent images are designed. For example, *W* can be chosen as the first-order discrete gradient operator (Rudin et al. 1992). However, these given linear operators are not optimal for all the images. Then some dictionary leaning methods that were adapted to the reference images were proposed to improve the image restoration quality, e.g., the so-called K-SVD method (Donoho and Elad 2003; Elad and Ahron 2006; Mairal et al. 2007) and the data-driven tight frame method (Cai et al. 2014). The data-driven tight frame method outperforms the K-SVD method in terms of computational efficiency. These adaptive methods could usually provide better sparse approximations by deriving adaptive discrete dictionaries or framelets from the input reference images.

The multiplicative problem can be converted into an additive one by taking the log of both sides of (1) (Shi and Osher 2008), i.e., the following so-called Log-TV model

2 $$\begin{aligned} \mathrm{log }f=\mathrm{log}u+\mathrm{log}\delta . \end{aligned}$$

Then the multiplicative noise removal scheme can be modeled by penalizing the sparse transform of $\mathrm{log}u$ instead of *u* based on the noisy observation $\mathrm{log }f$. Motivated by the data-driven tight frame scheme for additive noise removal (Cai et al. 2014), we propose the adaptive tight frame approach for multiplicative noise removal in this paper. We first construct the adaptive wavelet tight frame based on the logarithmic transformed given image and then recover the image of interest by solving a wavelet balanced approach using the constructed adaptive tight frame system. We perform this adaptive regularization method for images contaminated by speckle noise and the simulation results suggest that the proposed adaptive tight frame method improves the image restoration quality especially reducing the artifacts compared with the traditional non-adaptive one.

The reminder of this work is arranged as follows: the definitions and constructions of the wavelet tight frame are provided in section “Construction of the wavelet tight frame”. Then the scheme of learning the adaptive tight frame is given in the following section “Adaptive framelet”. We present the adaptive framelet algorithm for multiplicative noise removal in section “Multiplicative noise removal method based on adaptive tight framelet”. In Section “Numerical results”, the representative simulation results are reported. Finally, this work briefly concludes in section “Conclusion”.

## Construction of the wavelet tight frame

###  1D discrete wavelet tight frame

In this section, we first introduce the construction of the one-dimensional wavelet tight frame, i.e., its decompose and reconstruction. The corresponding 2D framelet is obtained by the tensor product of 1D framelets. More details can be seen in (Dong and Shen 2010; Chan and Shen 2003; Chan et al. 2004; Shen 2010).

A redundant tight frame in $\mathbb {R}^N$ is a sequence $\{x_i\}_{i=1}^{M}$ that satisfies

3 $$\begin{aligned} \sum _{i=1}^{M}|\langle x_i,g\rangle |^2=\Vert g\Vert _2^2,\quad \forall g\in \mathbb {R}^{N}. \end{aligned}$$

Actually, redundant tight frame ($M\ge N$) defined above is a generalization of orthogonal basis in $\mathbb {R}^N$, where $M=N$ and $\{x_i\}_{i=1}^{N}$ are linearly independent. There are two operators associated to the wavelet tight frame, i.e., analysis operator and synthesis operator. The analysis operator *W* is written as

$$\begin{aligned} W = [x_1,~x_2,~\ldots ,~x_M]^T. \end{aligned}$$

For a signal *g*, $Wg=\{\langle g,x_i\rangle \}_{i=1}^{M}$ denote the wavelet coefficients which are the inner products of $x_i$ and *g*. The synthesis operator denoted by $W^T$ is used to synthetize the the wavelet coefficients *s* by $W^Ts=\sum _{i=1}^M s(i) x_i$. Then the identity (3) is equivalent to

$$\begin{aligned} W^TW=I_{N}, \end{aligned}$$

where $I_N:\mathbb {R}^N\rightarrow \mathbb {R}^N$ denotes the identity operator.

Multi-resolution analysis (MRA) can be used to construct wavelet tight frame that associated with a so-called refinement mask $h_{0}$ and a class of MRA-based wavelet tight framelets are constructed (Dong and Shen 2010; Chan and Shen 2003; Chan et al. 2004; Shen 2010). For a signal $g\in \mathbb {R}^N$, the discrete wavelet operator *W* associated with filters $\varvec{h}=\{h_{0},h_{1},\ldots , h_{r-1}\}$ is defined as follows

4 $$\begin{aligned} W(\varvec{h}):~g\in \mathbb {R}^N\rightarrow \left[ \begin{array}{c} h_0*g\\ \vdots \\ h_{r-1}*g\\ \end{array}\right] \in \mathbb {R}^{rN}, \end{aligned}$$

where $*$ denotes the filtering procedure. $h_0$ denotes the low-pass filter which satisfies $\sum _j h_0(j)=1$, and $h_i$, $i=1,\ldots ,r-1$ satisfying $\sum _j h_i(j)=0$ is called high-pass filters usually. The multi-level wavelet operator can be obtained by using this one-level wavelet $W(\varvec{h})$ recursively to the low-pass coefficients. More details can be seen in (Chan and Shen 2003; Chan et al. 2004). The so-called perfect reconstruction property, i.e., $W(\varvec{h})^TW(\varvec{h})=I_N$ is equivalent to the following Unitary Extension Principle condition (Han et al. 2011):

5 $$\begin{aligned} \sum _{i=0}^{r-1}\sum _{l\in \mathbb {Z}}h_i(m+l)h_i(l)=\delta _m,\quad \forall m\in \mathbb {Z}. \end{aligned}$$

Here $\delta _m=1$ if $m=0$ and $\delta _m=0$ otherwise.

## Adaptive framelet

Choosing a fixed redundant system that performs well for all the images is rather difficulty, the dictionary learning approaches that are adapted to the images have been explored recently (Donoho and Elad 2003; Elad and Ahron 2006; Mairal et al. 2007; Cai et al. 2014). The well-known K-SVD method (Elad and Ahron 2006; Mairal et al. 2007) is such a representative work with the advantage of better approximating images with abundant textures compared with the non-learning schemes. Although K-SVD method outperforms the traditional non-learning ones, the poor computational efficiency results in the difficulty in practical application. Recently, Cai et al. (2014) proposed the data-driven tight frame construction scheme. On one hand, this designed adaptive tight frame scheme reduces the artifacts which usually exists in the images processed by the fixed wavelet tight frames, on the other hand, the minimization problem of learning the adaptive wavelet tight frame is high-efficiency compared with the K-SVD learning scheme. This adaptive framelet method has been used in CT image reconstruction (Zhou et al. 2013) and seismic data processing (Liang et al. 2014) etc. Next, we will first present the method of constructing the adaptive wavelet tight frame.

Let $\varvec{H}=\{H_i\}_{i=0}^{R-1}$ denote a family of two-dimensional filters. Then the 2D wavelet transform operator can be defined as

$$\begin{aligned} W(\varvec{H}):~g\in \mathbb {R}^{N^2}\rightarrow \left[ \begin{array}{c} H_0*g\\ \vdots \\ H_{R-1}*g\\ \end{array}\right] \in \mathbb {R}^{RN^2}. \end{aligned}$$

Here, we use $*$ to denote the filtering of two 2D arrays.

In Cai et al. (2014), the data-driven tight frame construction scheme was introduced in order to obtain better sparse representation of the given signal *g* by solving

6 $$\begin{aligned} \min \limits _{s,\varvec{H}}\Vert s-W_{a}(\varvec{H})g\Vert _{2}^{2}+\mu ^{2} \Vert s\Vert _{0}, \quad \mathrm{s.t. }\quad W_{a}(\varvec{H})^{T}W_{a}(\varvec{H})=I_{N^2}, \end{aligned}$$

where $W_{a}$ denotes the adaptive wavelet tight frame that satisfies $W_{a}^{T}W_{a}=I$. The first term in (6) is to ensure that the coefficients *s* are close to $W_{a}g$, and the second term is to make the coefficients *s* be sparse. Then adaptive 2D filters $\varvec{H}=\{H_i\}_{i=0}^{R-1}$ and the approximated sparse coefficients *s* can be obtained by solving (6). Based on the alternating minimization principle, (6) can be solved by solving two sub-problems, i.e.,

7 $$\begin{aligned} \min \limits _{s}\Vert s-W_{a}(\varvec{H})g\Vert _{2}^{2}+\mu ^{2} \Vert s\Vert _{0}, \end{aligned}$$

8 $$\begin{aligned} \min \limits _{\varvec{H}}\Vert s-W_{a}(\varvec{H})g\Vert _{2}^{2}, ~~\mathrm{s.t. } ~~W_{a}(\varvec{H})^{T}W_{a}(\varvec{H})=I_{N^2}. \end{aligned}$$

Observing that (7) is used to learn the adaptive tight frame based on the given image *g*. Obviously, the analytical solution of the *s*-subproblem (7) can be obtained via hard thresholding. More details about hard thresholding can be seen in Blumensath and Davies (2009, 2010). The second sub-problem (8) is a complex non-convex minimization problem with the quadratic constraints that can be simplified as

9 $$\begin{aligned} \sum _{i=0}^{R-1}\sum _{n\in \mathbb {Z}^2}H_i(k+n)H_i(n)=\delta _k,\quad \forall k\in \mathbb {Z}^2, \end{aligned}$$

where $\delta _k=1$ if $k=(0,0)$ and $\delta _k=0$ otherwise Han et al. (2011). Then the wavelet tight frame can be obtained by solving the following minimization problem with orthogonal constraints:

10 $$\begin{aligned} \min \limits _{\varvec{H}}\Vert s-W_{a}(\varvec{H})g\Vert _{2}^{2}, ~~\mathrm{s.t. } ~~\langle H_{i},H_{j}\rangle =\frac{1}{r^{2}}\delta _{i-j},~~0\le i,j\le R-1. \end{aligned}$$

Here $R=r\times r$ is also the support of $H_i,i=0,\ldots ,R-1$. This problem (10) can be solved exactly via the singular value decomposition (SVD) (Cai et al. 2014; Zou et al. 2006). Concretely, partitioning the coefficient vector *s* into $r^{2}$ vectors, denoted by $s_{i}\in \mathbb {R}^{N^2\times 1}, i=1,2,\ldots r^{2},$ corresponding to the coefficient obtained from the filter $H_{i}$. Define

11 $$\begin{aligned} \tilde{S} &= \left[ \begin{array}{ccc} s_{0}(1) &{} \ldots &{} s_{r^{2}-1}(1)\\ \vdots &{}\vdots &{} \vdots \\ s_{0}(N^{2}) &{} \ldots &{} s_{r^{2}-1}(N^{2}) \end{array} \right] \in \mathbb {R}^{N^2\times r^{2}} \nonumber \\ \tilde{G}&= [g_1,g_2,\cdots g_{N^{2}}]\in \mathbb {R}^{r^{2}\times N^2} \nonumber \\ \tilde{H} &= [H_{0},H_{1},\ldots ,H_{r^{2}-1}]\in \mathbb {R}^{r^{2}\times r^{2}}, \end{aligned}$$

where $g_i$, $i=0,\ldots ,N^2-1$, denotes the partition of *g* corresponding to the filter size *r*. Then the minimization (10) can be solved by solving the following problem

12 $$\begin{aligned} \max _{\tilde{H}} \mathrm {Tr}(\tilde{H}\tilde{S}^T\tilde{G}^{T}), ~s.t. ~\tilde{H}^{T}\tilde{H}=\frac{1}{r^{2}}I_{r^{2}}. \end{aligned}$$

### **Theorem 1**

Zou et al. (2006)* Let*$C_{m\times r}$*denotes matrix with rank r and the SVD decomposition of matrix*$A_{m\times r}$* is*$A=UDQ^{T},$* Then*$C_{*}=UQ^{T}$* is the solution of the following constrained maximization problem:*

13 $$\begin{aligned} \mathrm{max}_{C}\mathrm{Tr}(C^{T}A), ~s.t. ~ C^{T}C=I_{r^{2}}. \end{aligned}$$

Let $A=\tilde{S}^T\tilde{G}^{T}=UDQ^{T}$. Then by Theorem 1, the $W_{a}$-subproblem can be obtained by the following equation

14 $$\begin{aligned}{}[H_0,H_1,\ldots ,H_{r^{2}-1}]=\frac{1}{r}QU^{T}. \end{aligned}$$

## Multiplicative noise removal method based on adaptive tight framelet

The wavelet based sparse representation methods for Gaussian noise removal can be usually summarized as

15 $$\begin{aligned} s^{*}=\mathrm{arg}\min \limits _{s}\frac{1}{2}\parallel W^{T}s-f\parallel ^{2}_{2}\,+\,\frac{\tau }{2}\parallel (I-WW^{T})s\parallel ^{2}_{2}\,+\, \lambda \Vert s\Vert _{1}. \end{aligned}$$

Then the recovered image $u^{*}$ approximates $W^{T}s^{*}$. Here, the second term $\frac{\tau }{2}\parallel (I-WW^{T})s\parallel ^{2}_{2}$ is used to balance the distance between the coefficient *s* and the corresponding recovered signal $W^{T}s$, so three categories are distinguished by different values of $\tau$. When $\tau =0,$ the minimization (15) is called the synthesis based approach (Cai et al. 2009; Starck et al. 2005). Many effective iterative algorithms have sprung up to overcome the computational difficulty caused by the non-differentiable regularization term, e.g., split Bregman method (Goldstein and Osher 2009; Cai et al. 2010), alternating direction method (Afonso et al. 2010), forward-backward algorithm (Combettes and Pesquet 2007), primal-dual algorithm (Chan et al. 1999), etc. When $\tau =\infty ,$ the model (15) is just the analysis based approach (Daubechies et al. 2004). When $0 < \tau <\infty$, the above model is called a balanced approach (Cai et al. 2009; Chan et al. 2003). The three methods are equivalent if the transform operator *W* is orthogonal.

In this work, we will take the balanced approach for avoiding the multiple iterations produced in solving analysis based approach so as to reduce the computing consuming. The balanced approach yields the restoration result that balanced the sparsity of the associated tight framelet coefficients and the regularity of the recovered result. The degenerated image can be recovered by the following formula based on the wavelet tight framelet $W(\varvec{H})=W(H_{0},H_{1},\ldots ,H_{r^{2}-1})$ balanced approach

16 $$\begin{aligned} z=W^{T}(T_{\lambda }(W(\mathrm{log} f))), u=\mathrm{exp}(z). \end{aligned}$$

That is the following algorithm

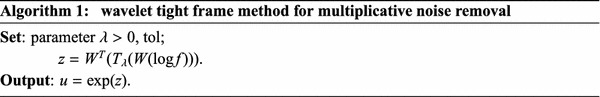



The constructed adaptive framelets were used in Gaussian noise removal and demonstrated their superiority in terms of image restoration quality compared with the corresponding non-adaptive ones (Cai et al. 2014) and improved computational efficiency compared with the K-SVD learning method. In this section, we will generalize the adaptive framelet method to multiplicative noise removal by considering the multiplicative denoising problem in log-domain. We convert multiplicative denoising problem into additive noise based one in log-domain. Then the logarithmic adaptive tight framelet constructed based on the noisy image in the log-domain, i.e., $\mathrm{log}f$ instead of *f*, is used for sparse representation so as to improve the recovered images quality. We summarize the adaptive tight framlet balanced approach for denoising the degenerated images contaminated by multiplicative noise as the following Algorithm 2.

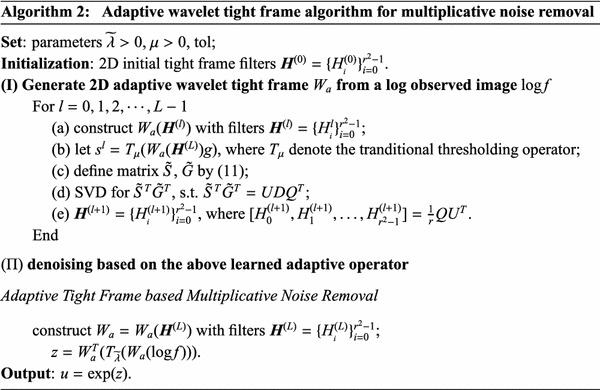



## Numerical results

This section will illustrate the restoration results by wavelet based multiplicative noise removal approaches. Furthermore, the superiority of the proposed adaptive wavelet based scheme (Algorithm 2) will be illustrated here through simulations on frequently-used test images “lenna” and “barbara”. The following peak signal to noise ratio (PSNR) is used to evaluate the recovery quality

17 $$\begin{aligned} \mathrm{PSNR}=20\mathrm{log}10\frac{255}{\frac{1}{MN}\Vert x-x_0\Vert _2}, \end{aligned}$$

where *x* and $x_{0}$ respectively denotes the recovered image and the original image with dimension $M\times N$. Larger PSNR value usually means better image restoration quality. We perform all the simulations on the PC with 2.9HZ and 64-bit operator system in the circumstance of 2009 MATLAB.

### *Example 5.1*

**Fig. 1:**
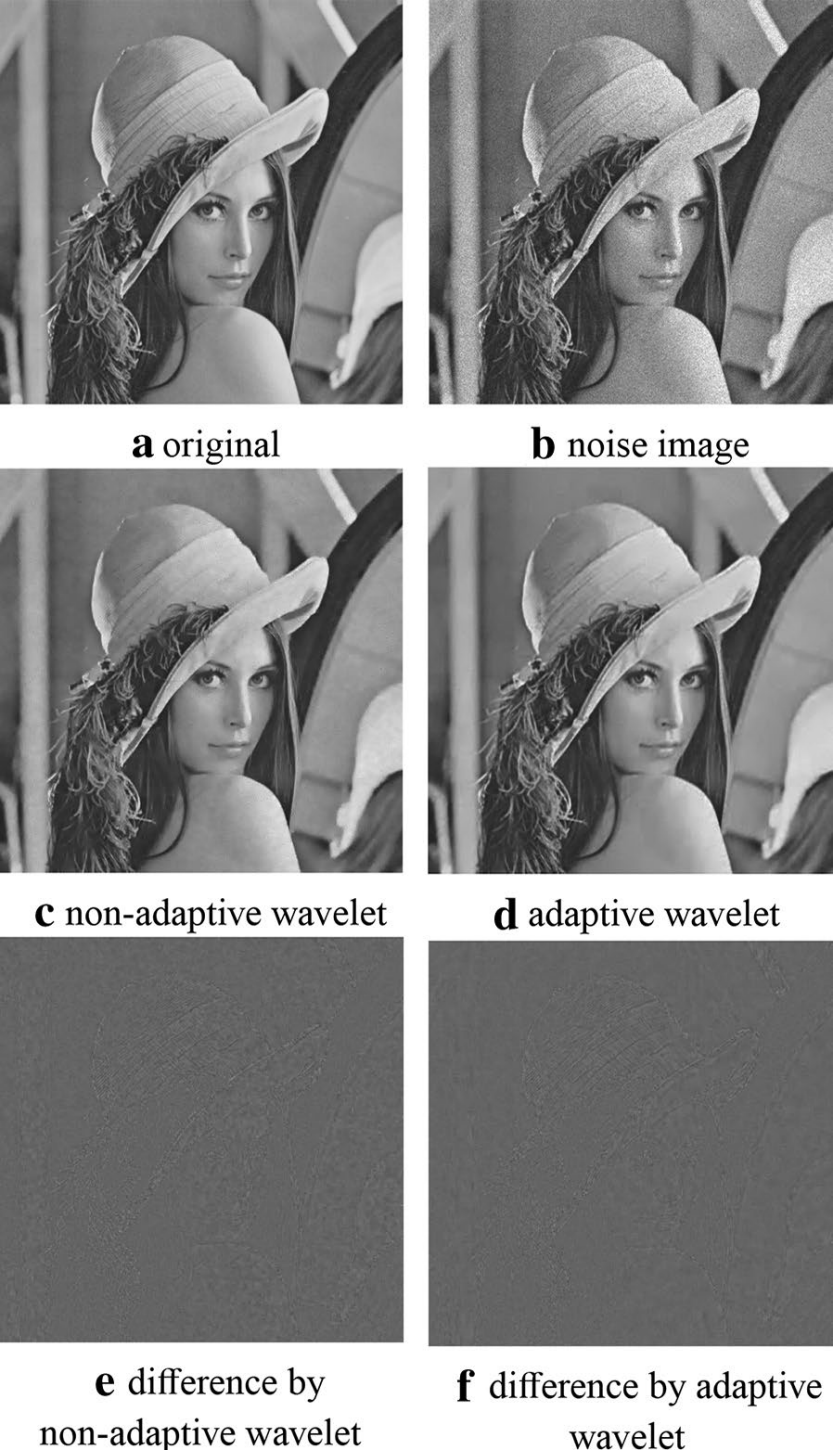
Denoised results for polluted “lenna” by adaptive wavelet tight frame and non-adaptive wavelet tight frame. **a** Denotes the original image and **b** is the noisy image. **c**, **d** are respectively the recovered images by adaptive wavelet tight frame method and the corresponding non-adaptive one with $8 \times 8$ Haar wavelet. **e** = **a**–**c** and **f** = **a**–**d** are the corresponding difference images

Figure 1 shows the recovered results by non-adaptive wavelet tight framelet and adaptive wavelet tight framelet approach based on the ground truth image “lenna” (Fig. 1a). “lenna” was polluted by multiplicative speckle noise with noise variance $\sigma =0.1$ (see Fig. 1b). Note that we can directly use the noisy image, i.e., Fig. 1b, as the reference image to learn the adaptive wavelet tight framelet operator. Figure 1c, d respectively illustrate the reconstructed images by non-adaptive wavelet framelet and adaptive wavelet framelet with the $8\times 8$ Haar wavelet as the initial filter. The difference images are respectively illustrated in Fig. 1e,f. It can be seen that adaptive framelet approach (Fig. 1d) can suppress artifacts and capture more details than the corresponding non-adaptive one (Fig. 1c), which also can be seen in difference images via comparing Fig. 1f by adaptive framelet approach with Fig. 1e by non-adaptive method. The PSNR values listed in Table 1 also reflect that adaptive framelet approach yields better recovered results than the corresponding non-adaptive one. In Table 1, we also compare the results by adaptive tight framelet approaches in terms of different initial filter sizes and filter types with the results by corresponding non-adaptive ones. Data in Table 1 demonstrates that adaptive tight framelet approaches defeat the corresponding non-adaptive ones with respect to image restoration quality evaluated by PSNR. Generally speaking, larger filter size means better restoration quality in terms of the tested filter size. The computation time for recovering a $256 \times 256$ image by a $3 \times 3$, $7 \times 7$, and $15 \times 15$ adaptive tight frame is respectively about 0.16, 1.48 and 14.75 s, which is more excellent than the K-SVD in terms of efficiency. Here and in the following test, the parameters used for learning the adaptive wavelet framelet and thresholding denoising follow the selection rule provided in Cai et al. (2014), that is $\alpha =5.1\sigma$ and $\widetilde{\lambda }=2.6\sigma$.

**Table 1:** Denoised results for polluted “lenna” by adaptive wavelet tight frames and the corresponding non-adaptive ones in terms of different filter sizes

Initial tight frame	Filter size	Non-adaptive algorithm	Adaptive algorithm
	$2 \times 2$	31.25	31.54
Haar wavelet	$4 \times 4$	32.91	33.89
	$8 \times 8$	33.16	34.64
	$2 \times 2$	31.26	31.45
Local DCT	$4 \times 4$	33.57	33.85
	$8 \times 8$	34.32	34.68
	$3 \times 3$	32.07	33.26
Linear framelet	$7 \times 7$	33.12	34.63
	$15 \times 15$	33.32	34.77

### *Example 5.2*

**Fig. 2:**
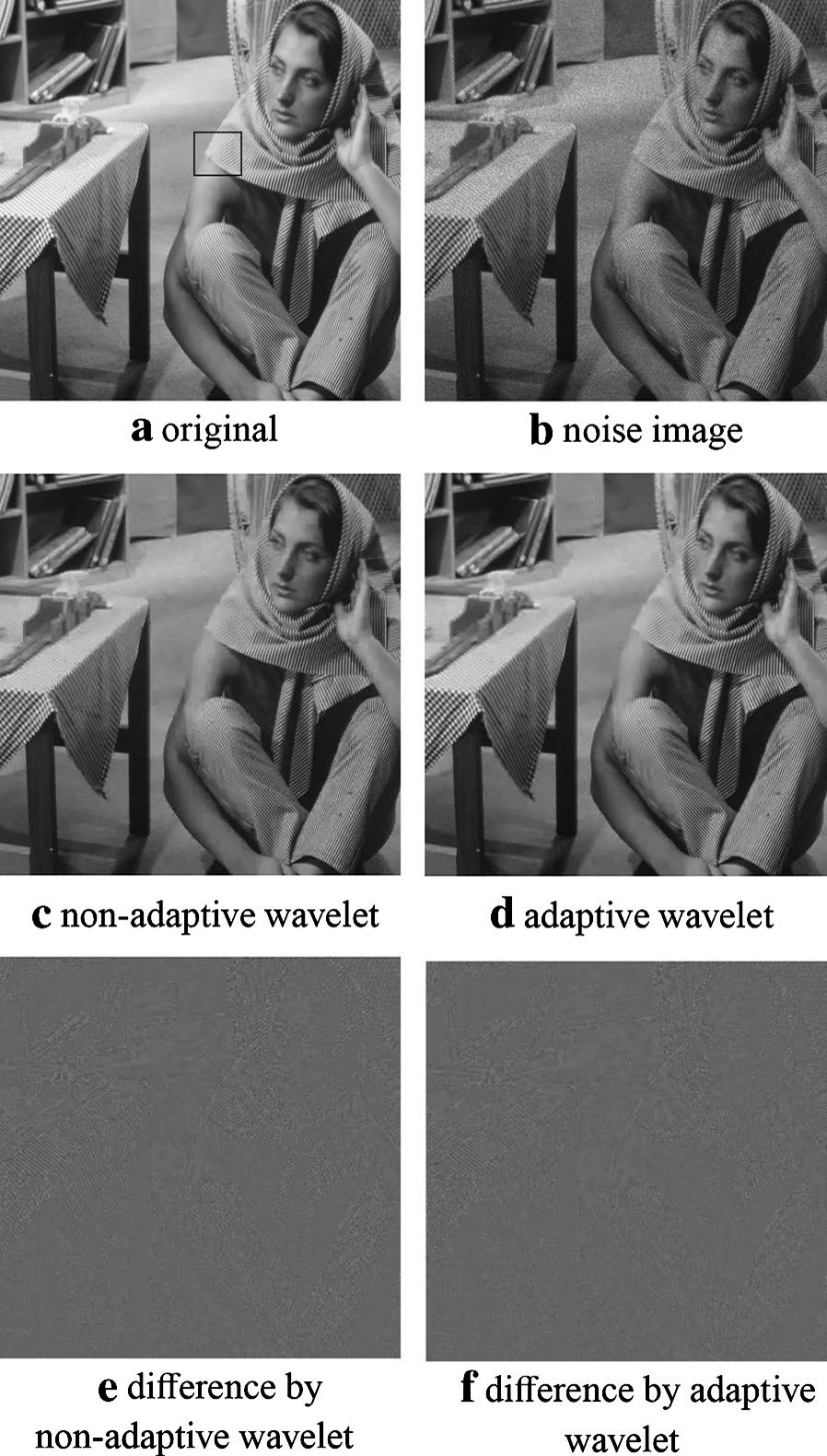
Denoised results for polluted “barbara” by adaptive wavelet tight frame and non-adaptive wavelet tight frame. **a** is the original image and **b** is the noisy image. **c**, **d** are respectively the recovered images by adaptive wavelet tight frame method and the corresponding non-adaptive one with$8 \times 8$ Haar wavelet. **e **=** a**–**c** and **f **=** a**–**d** are respectively the difference image

**Fig. 3:**
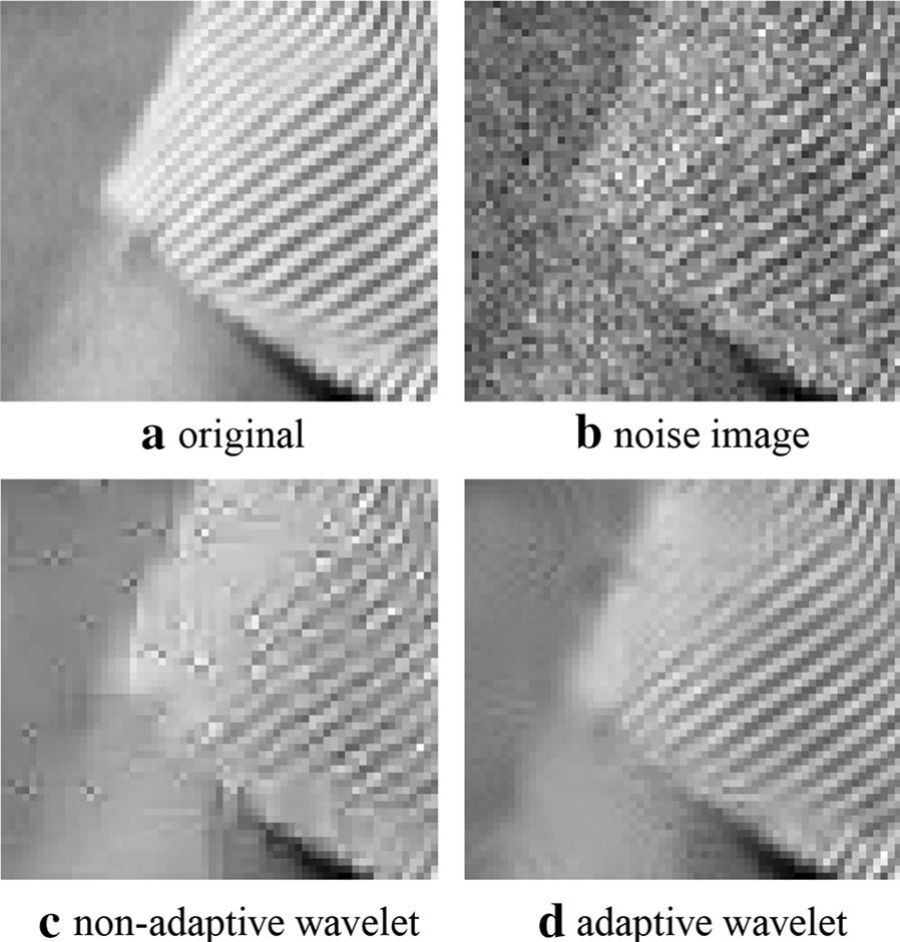
The zoomed denoised results for “barbara”, **a** is the original zoomed image and **b** is the zoomed noisy image. **c**, **d** are respectively the zoomed recovered images by adaptive wavelet tight frame method and the corresponding non-adaptive one

“barbara” (Fig. 2a) is usually used to assess the ability of catching textures for different algorithms owing to its abundant textures. Figure 2b is the degenerated “barbara” with multiplicative speckle noise variance $\sigma =0.1$. Figure 2c, d respectively present the recovered images by non-adaptive wavelet framelet and adaptive wavelet framelet with the $8\times 8$ Haar wavelet as the initial filter. Figure 2e, f are the corresponding difference images respectively. Obviously, adaptive framelet approach (Fig. 3d) has the advantages in preserving textures and suppressing artifacts compared with the non-adaptive scheme (Fig. 3c) from the zoomed part of “barbara” (Fig. 3). Repeatedly, we compare the results by adaptive tight framelet approaches in terms of different initial filter sizes and filter types with the results by corresponding non-adaptive ones (see Table 2).

**Table 2:** Denoised results for polluted “barbara” by wavelet tight frame method and adaptive wavelet tight frame in terms of different filter sizes

Initial tight frame	Filter size	Non-adaptive algorithm	Adaptive algorithm
Haar wavelet	2 $\times$ 2	29.44	29.55
	$4 \times 4$	30.18	31.39
	8 $\times$ 8	30.46	32.72
Local DCT	2 $\times$ 2	29.37	29.48
	$4 \times 4$	31.27	31.55
	8 $\times$ 8	32.32	32.71
Linear framelet	3 $\times$ 3	29.91	30.94
	$7 \times 7$	30.63	32.41
	$15 \times 15$	31.05	32.67

Table 3 lists the SNR results of the adaptive B-Spline wavelet framelet method in terms of larger speckle noise with variance $\sigma =0.2$. From Table 3, we can see that our algorithm can also obtain better results than the corresponding non-adaptive ones.

**Table 3:** Larger noise removal results by our proposed adaptive wavelet tight frame and the corresponding non-adaptive one

Image	Filter size	Non-adaptive algorithm	Adaptive algorithm
	$3 \times 3$	26.64	27.58
Lenna	$7 \times 7$	26.96	28.96
	$3 \times 3$	24.75	25.81
Barbara	$15 \times 15$	24.87	27.95

## Conclusion

In this paper, we have generalized the adaptive tight frame methods to multiplicative noise removal problem. We learn the image-specific wavelet tight framelet based on the logarithmic transformed image by using SVD based explicit formulas. Numerical experiments demonstrate that the derived adaptive tight frame balanced based regularization method improves the image restoration quality compared with the corresponding non-adaptive one not only in enhancing PSNR value but also in preserving fine image features.
